# Pattern of dermatoses in Wolaita zone prison setting: a call for improved dermatology services

**DOI:** 10.3389/fmed.2025.1451089

**Published:** 2025-02-18

**Authors:** Abraham Getachew Kelbore, Efa Ambaw Bogino, Aldo Morrone

**Affiliations:** ^1^Department of Dermatology, College of Health Sciences and School of Medicine, Wolaita Sodo University, Wolaita Sodo, Ethiopia; ^2^International Institute Social, Medical, Anthropological Sciences (IISMAS), Rome, Italy

**Keywords:** skin diseases, prevalence, dermatology service, prison, Ethiopia

## Abstract

**Background:**

Skin diseases are not uncommon among prisoners, primarily due to confined living conditions, limited access to proper hygiene facilities, and higher rates of skin-to-skin contact. The study aims to describe the skin disease spectrum among prison inmates Wolaita zone, southern Ethiopia.

**Methods:**

A cross-sectional study was conducted at the Wolaita zone prison to determine the spectrum of skin diseases among the prison inmates from January 1 to February 30, 2020. Every inmate with skin complaints underwent a comprehensive skin examination, and a detailed history of their skin-compliant was documented. The diagnosis primarily relied on clinical assessment by dermatologists. The data collected from paper-based abstraction sheets was entered into EpiData entry forms twice to ensure accuracy. A descriptive analysis was performed such as frequencies, mean, standard deviation and median. The statistical significance was set at 0.05.

**Result:**

Out of the 418 prison inmates who took part in the study, 223 (53.3%) were found to have skin disorders. The vast majority of the participants, specifically 381 (91.1%), were male. The age range of the participants varied from 17 to 60 years old, with an average age of 29.29 years ± 9.08 years. Skin infections were identified as the most prevalent type of skin disease, with 113 patients (50.67%) affected. Among the skin infections, fungal infections and scabies infestations were the most common, accounting for 41 cases (18.4%) and 37 cases (8.85%) respectively. In terms of inflammatory skin problems, 50 cases (11.9%) of Eczematous skin diseases were diagnosed. Within this category, Atopic Eczema and Nummular eczema accounted for 19 (4.5%) and 8 (1.9%) cases, respectively. Notably, a case of Leprosy was also diagnosed and linked to treatment within this prison.

**Conclusion:**

In this study, infectious skin diseases and manageable inflammatory skin diseases are commonly diagnosed as dermatoses among prison inmates at Wolaita zone Prison. The inmates have the right to the best of health, including skin health, so health professionals posted to prison services must be trained to diagnose and manage skin disorders in prisons.

## Background

Skin diseases among prison inmates have been documented in both developed and developing countries, highlighting significant health challenges, particularly related to skin conditions (1, 2). Prisons have long been a feature of society, serving correctional centres for lawbreakers (3). In developing countries, the infrastructure of prisons is usually in poor condition; as a result, skin diseases are quite common among prisoners due to inadequate housing conditions, overcrowding, hot and humid environments, lack of ventilation, poor nutrition, poor personal hygiene and close living quarters, exacerbate the prevalence and severity of skin diseases (4–6). This has a significant public health impact because the vast majority of people in prison will return to the community, further burdening the existing healthcare facilities. The length of imprisonment has also been demonstrated to have a substantial association with skin infections (5). All of these contribute to further stress and psychosocial damage, which leads to neglect of one’s health (1). Studies conducted in different countries have shown varied patterns of skin diseases among prison inmates in Cameron (7), prison inmates in India (8), and Nigeria (4), female prison inmates in Turkey (9), and prison inmates in Southern Lazio, Italy (10). However, it is difficult to compare the morbidity of skin diseases in different countries because the diagnostic methods are inconsistent. Some of the cases are diagnosed based on disease history and clinical findings (7), histopathological examination (4), and diagnosis by skin disease specialists without sufficient details (9, 10).

Due to heredity, environmental factors, hygiene standards, and social conventions, skin illness patterns differ from country to country and even region to region within the same country (11, 12). In Ethiopia, there is a limited dermatology service at prison clinics and getting through a referral system. There is a scarcity of studies on the profile of skin diseases among prison inmates. Also, there is no data regarding patterns of skin diseases among prison inmates in the Wolaita zone, southern Ethiopia. The World Health Organization has suggested reducing any “avoidable or unfair” health differences, stating that prisoners are entitled to have equal access to health services (13). Therefore, this study helps to document the spectrum of skin diseases among prison inmates of Wolaita zone seen at the prison clinic in Wolaita Sodo for the study period availing a service within the prison.

## Aim

To describe the spectrum of skin diseases among prison inmates of Wolaita zone, southern Ethiopia, between January 1st to February 30th, 2020.

## Methods

### Study setting

The study was conducted in Wolaita zone prison in South Ethiopia. It is located 330 km away from the capital city, Addis Ababa. The prison consisted of a total of 1,656 inmates. A prison clinic provides outpatient and emergency services for prison inmates. The service is offered by two health officers, three BSc nurses, and two diploma nurses, and it offers 24-h service. There is no medical check-up before entering the prison. Skin diseases are managed by the health officer or BSc nurses in routine practice. When dermatology consultation is needed, the prisoners are referred to the Wolaita Sodo University comprehensive specialized Hospital dermatological clinic. The waiting period to get a dermatologist is 15–30 days because outpatient visits require prison staff to arrange a secure transfer to the hospital.

### Study design, study population, and sampling

This cross-sectional study design was performed from January 1st to February 30, 2020, in a Wolaita zone prison in Wolaita Sodo, southern Ethiopia. Prison inmates aged ≥18 years and who visited the prison clinic during the study period were the study population.

During the study, all inmates who visited the clinic were examined for skin diseases and dermatological conditions at work. All the inmates with dermatological compliant were subjected to a detailed cutaneous examination. A brief history of the skin complaints was elicited, further laboratory investigations were sent to Wolaita Sodo Comprehensive Specialized Hospital. Patients were counseled correctly and prescribed appropriate treatment.

### Data collection and quality control

#### Diagnosis and management of skin diseases

All prison inmates who present with skin conditions are managed by three Tropical dermatology professional specialists at the prison clinic. Diagnosis is made on clinical grounds as well as by laboratory support. Relevant laboratory tests include KOH tests, skin smear tests, and gram stain or histopathology, and other investigations as necessary.

A structured interview questionnaire was used in the local language once translated from the English version to Amharic and then back to English by different professional translators to ensure the consistency of the information.

Additional data was reviewed from the clinical examination cards of the prison inmates. The interview was conducted among the prison inmates at the prison clinic visit during the study period.

### Analysis and statistic

Data was double-entered from the paper-based abstraction sheets into EpiData software *(v4.2.0.0 for entry Epi-Data Association, Odense, Denmark)*. A descriptive analysis of frequencies, mean, standard deviation and median were perform. Categorical variables about skin diseases, age, sex, and residence were compared using the Chi-square test. Continuous variables like age were compared and presented using the appropriate means. Levels of significance were set at 5%.

### Ethics considerations

Ethics approval was obtained from the Ethical Review Committee College of Health Science and Medicine, Wolaita Sodo University (CARD 865/869/12). Accordingly, Permission for the study was secured from the Wolaita zone prison administrator and Zonal health department before data collection. Prison inmate Patient identification variables were not used in the study. The studies do not inflict harm on or expose prisoners to unnecessary risk because of examining prisoners and interviewing them. Written Informed consent was obtained during the interview. When interviews and physical examinations were completed, those inmates who had the problem were treated accordingly.

## Results

Of the total of 422 prison inmates chosen for the study, 418 participated, resulting in a response rate of 99%. Of the sampled inmates, only 223 (53.3%) presented with skin problem complaints, while 381 (91.1%) being male and 37 (8.9%) being female. The age of the participants ranged from 17 to 60 years, with an average age of 29.29 ± 9.08 years. More than half of the inmates (83.7%) stayed in rural environments while less than a fifth (16.3%) lived in urban areas before getting in the prison. Additionally, 343 (82.1%) were literate. Sociodemographic characteristics are shown in Table 1.

**Table 1:** Socio-demographic characteristics of prisoner inmates (*N* = 418) Wolaita zone prison, southern Ethiopia, 2020.

Variables		Frequency	Percent (%)
Sex	Male	381	91.1
	Female	37	8.9
Residence before imprisonment	Rural	350	83.7
	Urban	68	16.3
Able to read and write	Yes	343	82.1
	No	75	17.9
Highest grade completed	Primary (1 to 8)	193	46.2
	Secondary (9 to 12)	124	29.7
	Tertiary	2	0.5
	Diploma	10	2.4
	Degree and above	14	3.3
Age category	18–27 yrs	199	47.6
	28–37 yrs	149	35.6
	38–47 yrs	44	10.5
	≥48 yrs	26	6.2

Among 223 new cases, skin infections were the most common skin disease 113 (50.67%). Of these fungal infections were the most common type of skin infection, followed by infestation, bacterial infection and viral infections. Scabies was the most common contagious skin disease observed from ecto parasitic diseases or infestations. From non-infectious disorder, Eczema was identified as the most common disorder among prisoners, followed by Pilosebaceous, pigmentary disorders, and other miscellaneous skin diseases. A new confirmed case of leprematous leprosy case was diagnosed among chronic bacterial skin diseases (Table 2).

**Table 2:** Spectrum of skin diseases among prison inmates (*N* = 418) Wolaita zone prison, southern Ethiopia, 2020.

Sr.N	Diseases category	Frequency (N)	Percentages (%)
1	**Fungal infections**	**41**	**9.8**
	Tinea corporis	9	2.2
	Tinea manu	3	0.7
	Onychomycosis	1	0.2
	Tinea pedis	4	1
	Pityriasis versicolor	24	5.7
2	**Bacterial infections**	**29**	**6.8**
	Impetigo	**6**	**1.4**
	Folliculitis	**10**	**2.4**
	Ecthyma	**5**	**1.2**
	Carbuncle	**5**	**1.2**
	Furuncle	**2**	**0.4**
	Leprosy	**1**	**0.2**
3	**Viral infections**	**6**	**1.4**
	Cutaneous wart	4	1
	Mulloscum contagisum	2	0.4
4	**Infestations**	**37**	**8.85**
	Scabies	37	8.85
5	**Eczema**	**50**	**11.9**
	Atopic dermatitis	19	4.5
	Nummular eczema	8	1.9
	Seborrehic dermatitis	3	0.7
	Allergic contact dermatitis	5	1.2
	Lichen Simplex chronicus	15	3.6
6	**Papulosquamous disorders**	**6**	**1.4**
	Psoriasis	3	0.7
	Lichen planus	3	0.7
	Pityriasis rosea	1	0.2
7	**Pilosebaceous disorders**	**13**	**3.1**
	Acne vulgaris	12	2.9
	Rosacea	1	0.2
8	**Drug Eruptions**	9	2
	Urticaria	9	2
9	**Pigmentary disorders**	**13**	3.1
	Vitiligo	8	1.9
	Melasma	5	1.2
10	**Miscellaneous diseases**	**19**	4.2
	Keloid	7	1.7
	Lichen sclerosus	2	0.4
	Alopecia areata	6	1.4
	Nevus	4	1

Bold values: indicate the sum in the category of the disease.

Age of participant, educational status, and residence before imprisonment were significantly associated with skin diseases [chi-square (*x^2^*) = 0.029, *x^2^* = 0.001 and *x^2^* = 0.02 respectively].

## Discussion

This is the first observational study that identified the spectrum of skin diseases among prison inmates in southern Ethiopia. Prisons are a suitable environment for many skin diseases. Inmates live in a dynamic equilibrium between prison and community. Hence, timely case detection and treatment of skin diseases is important to decrease the community’s spread, recurrences, complications, and disease burden (14).

In this study, the prevalence of infectious skin diseases was higher compared to the study conducted countries, such as Cameroun (7), India (8), Nigeria (5) and Turkey. Our research has shown that infectious skin diseases are the most frequent type, accounting for more than half of all cases (50.67%). This high prevalence suggests a combination of factors, including overcrowding, poor hygiene facilities, and compromised immune systems due to stress or pre-existing health conditions. These differences could be due to variations in environmental conditions, healthcare accessibility, cultural practices, and demographics across different regions. Additionally, socioeconomic factors, such as prison infrastructure and healthcare provisions, may affect the prevalence and management of skin diseases within correctional facilities.

The spectrum of skin diseases observed in this study differs from those reported in other studies study conducted in Taiwan by Jiesisibieke et al. (15), has reported eczematous skin diseases was higher followed by infectious diseases (15). However, in the current study cutaneous infectious diseases like, parasitic infestations and fungal and bacterial infections were prevalent. These disparities may be attributed to variations in geographic locations, sample size, study duration, study design, differences in the prison environment or, variations in the setup of prison clinics, and differences in the socioeconomic status of the populations studied.

The burden of infectious skin diseases and other chronic diseases, mental diseases, and cognitive disability is higher among prison inmates (16). This is associated with poor environment and hygiene safety considerations, and it might not be convenient for prison inmates to receive timely treatment.

The results of our study are consistent with findings from various other countries, where infectious skin diseases were identified as the most prevalent cases, accounting for 50.7% of all cases. Among these, 36.3% were attributed to fungal infections, 32.7% to scabies infestation, 25.7% to bacterial infections, and 5.3% to viral infections. This underscores the significant impact of infectious skin diseases on public health and emphasizes the need for effective prevention and treatment strategies.

In this study, scabies 8.85% is the most common ecto-parasitic contagious skin disease observed among prisoners. Our finding shows that the prevalence of scabies is low compared to studies conducted in Cameroon 32% (17) and Nigeria 12% (18). However, this study differs from others in that the prevalence of scabies was found to be higher study conducted in Italy and Poland prison (0.72 and 2.24% respectively) (10, 19). This could be attributed to the regular administration of ivermectin in this particular prison to prevent onchocerciasis, which may have contributed to the reduced prevalence. This may emphasize the need for integrated case management of skin neglected tropical diseases (Skin-NTDs) and adhering to appropriate managmentto combat the spread of diseases within prison environments.

The prevalence of pitriasis versicolor infections is the most common among fungal infections disorders. This is supported by studies conducted in Jimmu, India (20). In fact, in tropical countries, the prevalence of pityriasis versicolor is in between 30 and 40% (21). This can be attributed to factors such as overpopulation, misuse of topical steroids, humidity, hot weather, and also in developing countries, immunosuppression (HIV); all of which create favorable conditions for the growth and transmission of fungal infections.

In this study, there was a significant relationship between the participant’s age, educational status, and residence before imprisonment with the likelihood of developing skin diseases (*x^2^* = 0.029, *x^2^* = 0.001 and *x^2^* = 0.02 respectively). This implies that age substantially influences the prevalence and type of skin conditions observed within prison populations; from our study observed most of the prisoners identified with skin diseases were aged between 18 and 27 years, and the study is in line with a study conducted in Nigeria (22). In our study, most of prisoners have lower educational attainment before incarceration, which can affect their health literacy and understanding of skin care. Rural residence before enrollment in correction centers showed a significant number of skin disease this could be due to the lack of dermatological service at peripheral units for rural communities in the study area (23). Furthermore, in this study, we identified and connected with a patient suffering from Leprosy. This disease is often overlooked and can affect the peripheral nerves, leading to various complications. This discovery highlighted the importance of providing dermatological services at the prison level, benefiting the prisoners and contributing to the prevention and control of such neglected tropical skin diseases within the broader community.

According to Italian research, around 40% of patients with skin illnesses altered or discontinued treatment without consulting physicians due to a lack of understanding about COVID-19 (24). The findings suggested that the COVID-19 pandemic had a detrimental impact on skin health-related treatment. Furthermore, COVID-19 prevention challenges may lead to patients with chronic diseases being overlooked, especially in low- and middle-income countries (25). In prisons, healthcare professionals should maintain health services for prisoners with chronic diseases such as skin diseases through teledermatology since it is possible to use in the prison environment for multiple reasons like security and unnecessary mobilization of prison inmates (26).

The study was limited to prison inmates seeking treatment for skin diseases at a single prison in Wolaita Sodo, southern Ethiopia. This limitation should be considered as a potential drawback of our study.

## Conclusion

The prison environment is overcrowded and is suitable for contagious skin problems and other inflammatory skin diseases compared to the general population. However, there was no dermatology service for proper prevention and management in the prison where the study was conducted.

In this study, infectious skin diseases and manageable inflammatory skin diseases are commonly diagnosed as dermatoses among prison inmates at Wolaita zone prison. So, it is important to give due attention to the periodic screening of prison inmates by dermatologists for early prevention, management of infectious skin diseases and other skin diseases.

The inmates have the right to the best of health, including skin health, so health professionals posted to prison services must be trained in the diagnosis and management of skin disorders in prison settings.

## Data Availability

The raw data supporting the conclusions of this article will be made available by the authors, without undue reservation.
